# Oxidative DNA Damage and Zinc Status in Patients With Rheumatoid Arthritis in Duhok, Iraq

**DOI:** 10.7759/cureus.52860

**Published:** 2024-01-24

**Authors:** Wahid M Hassan

**Affiliations:** 1 Surgery, University of Duhok, Duhok, IRQ

**Keywords:** das, 8-ohdg, zinc, dna damage, rheumatoid arthritis

## Abstract

Background

In patients with rheumatoid arthritis, oxidative DNA damage is increased by deficient zinc levels as well as increasing disease activity. However, the relationship between zinc levels, disease activity, and oxidative DNA damage remains unclear. In this study, we investigated serum zinc levels and disease activity and their association with 8-hydroxy-2-deoxyguanosine (8-OHdG).

Methodology

This case-control study was conducted among rheumatoid arthritis patients (n = 264) and healthy individuals (n = 192). Oxidative DNA damage was assessed by measuring serum 8-OHdG using enzyme-linked immunosorbent assay. Colorimetry was used to measure serum zinc levels. Disease activity was assessed using the Disease Activity Score-28 (DAS-28) score.

Results

Significantly higher 8-OHdG levels (p < 0.00) were found in the test group compared to the control group. Moreover, significantly lower serum zinc levels (p < 0.001) were noted in patients with rheumatoid arthritis compared to the control group. In addition, higher 8-OHdG levels were found in patients with low serum zinc levels compared to those with normal mean serum zinc levels. Lower levels of DNA oxidative damage were found in patients with moderate and low disease activity compared to those with high disease activity. A significant negative correlation was noted between serum zinc levels and DAS-28 scores and oxidative DNA damage marker (r = - 0.30, p = 0.038 and r = - 0.26, p = 0.043, respectively), while a significant positive correlation was observed between body mass index and 8-OHdG (r = 0.22, p = 0.02) in healthy individuals.

Conclusions

High serum 8-OHdG levels and high disease activity with low mean serum zinc levels may indicate a high degree of oxidative DNA damage in patients with rheumatoid arthritis.

## Introduction

Rheumatoid arthritis is a progressive disease that is associated with significant morbidity and disability as the disease progresses. Oxidative stress is a pathogenic hallmark in patients with rheumatoid arthritis due to the high cellular production of reactive oxygen species (ROS) [1,2]. Although many studies have focused on the biomarkers of DNA damage, only a few human studies have assessed these biomarkers in the clinical management of the disease [3,4].

Zinc is essential for the optimal function of many proteins and enzymes intracellularly and plays a role in many cellular activities such as cell proliferation, preventing oxidative stress by defending against ROS production, cellular apoptosis, and DNA base strand damage repair. Zinc plays a pivotal role along with superoxide dismutase enzyme as a first-line defense against ROS. Moreover, the transcription factor p53 plays an important role as a response to oxidative DNA damage [5]. When plasma and/or cellular zinc is deficient, this may lead to oxidative stress, impaired binding activity of p53, and non-proper functioning of p53, interfering with p53 functions in the repair of damaged DNA [6]. Thus, zinc deficiency causes impaired DNA integrity through several mechanisms which may lead to DNA damage by oxidative stress directly and/or may impair the repair system of damaged DNA [7].

DNA damage may be a result of disturbances between cellular antioxidant networks and repair systems [8]. Previous studies have shown that lower zinc levels cause oxidative DNA damage as an independent risk factor [9]. A study with a larger sample size and more prospective and intervention aspects will provide additional information on this hypothesis and study the zinc supplementation effects in decreasing oxidative DNA damage, especially in patients with rheumatoid arthritis.

## Materials and methods

This case-control study was conducted among patients who attended the Center of Rheumatology in Duhok, Kurdistan region of Iraq, between February 1, 2023, and August 1, 2023. A total of 264 patients diagnosed with rheumatoid arthritis and 192 healthy individuals were included. The study protocol was approved by the Committee of Medical Ethics of Duhok Directorate of Health. The patients and controls were matched for sex, age, and body mass index (BMI). Inclusion criteria were having the disease for more than five years, not taking any drugs other than for rheumatoid arthritis, and not taking multivitamins or mineral supplements in the last six months. Exclusion criteria were smoking, alcohol consumption, recent infections, and pregnancy. Inclusion criteria for the control group were healthy individuals with no metabolic or endocrine diseases. Smokers or alcoholics and those taking multivitamins or mineral supplements during the last six months were excluded from the control group. Moreover, those with a family history of rheumatoid arthritis among first-degree relatives were also excluded from the control group. The control group individuals were selected from relatives of Azadi Teaching Hospital workers and the Center of Rheumatology in Duhok governorate. A systemic random sampling procedure was used to select cases and controls.

After an overnight fast, both patients and controls attended the center for examination. Blood samples were collected at 8 to 10 o’clock. Anthropometric analysis was done for height and weight. BMI was calculated as follows: weight (kg) divided by (height) in m^2^. The collected samples were put in plain tubes with no anticoagulant, allowed to clot for one hour, and then centrifuged for serum separation. The sera were then processed directly for serum zinc measurement, and the remained sera were put in tubes and stored (frizzed at -80 degrees) for measurement of 8-hydroxy-2-deoxyguanosine (8-OHdG) later on. Serum levels of zinc were measured calorimetrically by a spectrophotometer (Centeronic GmbH, Germany). Enzyme-linked immunosorbent assay was used to measure the serum level of 8-OHdG (Elabsciencecata/log number E-El-0028, USA). Participants were grouped into mild-to-moderate and severe DNA damage according to the cutoff point of 8-OHdG (4.0 ng/mL). Individuals with a serum 8-OHdG level of less than 4.0 ng/mL were classified as the mild-to-moderate DNA damage group, and those with a serum level of more than 4.0 ng/mL were considered the severe DNA damage group [10]. Subjects with serum zinc levels of more than 70 µg/dL up to 120 µg/dL were considered to have normal serum zinc levels and those having zinc levels between 50 µg/dL and 70 µg/dL were considered to have mild-to-moderate zinc deficiency, while individuals with serum zinc levels of less than 50 µg/dL were considered to have severe zinc deficiency [11]. The status of disease activity was evaluated using the Disease Activity Score-28 (DAS-28) assessed by an orthopedic specialist physician [12,13]. Patients were classified into high (DAS-28 >5.1), moderate (3.2 < DAS-28 ≤ 5.1), or low (DAS-28 ≤3.2) disease activity groups.

Statistical analysis

SPSS version 18.0 (SPSS Inc., Chicago, IL, USA) was used for data analysis. Mean ± SD was used to present descriptive statistics. Assessment of differences in serum analyte among different groups was done by unpaired Student’s t-test and independent t-test. The chi-square and analysis of variance tests were used to assess the significance of differences between groups. The correlation between 8-OHdG, age, BMI, and zinc level was assessed using Pearson’s correlation coefficients test. P-values ≤0.05 were considered statistically significant.

## Results

The general and laboratory characteristics of the study participants are illustrated in Table 1. There was no significant difference between patients and healthy individuals regarding age, sex, and BMI. A significant difference was found between patients and healthy individuals regarding mean ± SD of serum 8-OHdG (5.94 ± 2.8 ng/mL for patients and 3.59 ± 2.0 ng/mL for healthy individuals, p < 0.01). Lower mean serum zinc level was found in rheumatoid arthritis patients when compared to healthy individuals and the difference was statistically significant (67.2 ± 25.8 µg/dL and 84.2 ± 29.4 µg/dL, p < 0.01). Regarding disease activity, among rheumatoid arthritis patients, 52.7% had DAS-28 equal to or less than 3.2, 34.4% had DAS-28 of 3.2-5.1, and only 12.9% of patients had DAS-28 of more than 5.1. The prevalence of high DNA damage (8-OHdG level >4.0 ng/mL) was 83.7% in patients with rheumatoid arthritis compared to 28.6% in healthy individuals (p < 0.01).

**Table 1:** Baseline characteristics of rheumatoid arthritis patients and healthy individuals. 8-OHdG = 8-hydroxy-2-deoxyguanosine; BMI = body mass index; DAS-28 = Disease Activity Score-28

Characteristics	Patients (n = 264) (mean ± SD)	Healthy individuals (n = 192) (mean ± SD)	P-value
Age (years)	48.9 ± 8.2	47.5 ± 8.5	0.11
Male, n (%)	110 (41.6)	74 (38.5)	0.48
BMI	27.1 ± 4.6	29.5 ± 6.4	0.06
Zinc (µg/dL)	67.2 ± 25.8	84.2 ± 29.4	<0.01
DAS-28 (>5.1), n (%)	34 (12.9)	-	-
DAS-28 (3.2–5.1), n (%)	91 (34.4)	-	-
DAS-28 (<3.2), n (%)	139 (52.7)	-	-
8-OHdG (ng/mL)	5.94 ± 2.8	3.59 ± 2.0	<0.01
8-OhdG (>4.0 ng/mL), n (%)	221 (83.7)	55 (28.6)	<0.01

Table 2 shows the serum 8-OHdG levels stratified to different zinc levels in patients and healthy individuals. Mean serum 8-OHdG levels were higher in low serum zinc level groups (<70 µg/dL) when compared to normal zinc levels (>70 µg/dL) in both patients and healthy individuals (6.61 ± 3.6 ng/mL and 5.87 ± 2.91 ng/mL vs. 5.29 ± 2.84 ng/mL and 5.01 ± 2.7 ng/mL) (p = 0.021 and p = 0.069, respectively). A higher mean level of 8-OHdG was found in the severe zinc deficiency group (6.91 ± 3.88 ng/mL) in rheumatoid arthritis patients when compared to normal zinc levels (5.29 ± 2.84 ng/mL), and the difference was statistically significant (p = 0.04); however, this difference in mean 8-OHdG in healthy individuals group did not reach statistical significance (6.13 ± 3.5 ng/mL vs. 5.01 ± 2.7 ng/mL) (p = 0.08).

**Table 2:** Serum 8-OHdG stratified by the zinc level. **: Comparing normal zinc status with the marginal zinc deficiency group. *: Comparing normal zinc status with the severe zinc deficiency group. 8-OHdG = 8-hydroxy-2-deoxyguanosine

Zinc status (µg/dl)	N (%)	Mean ± SD	P-value
Patients			
Low (<70)	177 (67.1)	6.61 ± 3.6	0.021
Normal (>70)	87 (32.9)	5.29 ± 2.84	
Healthy individuals			
Low (<70)	99 (51.6)	5.87 ± 2.91	0.069
Normal (>70)	93 (48.4)	5.01 ± 2.7	
Patients			
Severe zinc deficiency	28 (10.6)	6.91 ± 3.88	*0.04, **0.2
Marginal zinc deficiency	149 (56.5)	5.9 ± 3.23	
Normal zinc level	87 (32.9)	5.29 ± 2.84	
Healthy individuals			
Severe zinc deficiency	31 (16.1)	6.13 ± 3.5	*0.08, **0.06
Marginal zinc deficiency	68 (35.5)	5.84 ± 2.93	
Normal zinc level	93 (48.4)	5.01 ± 2.7	

Table 3 illustrates the mean serum 8-OHdG level and zinc level according to the DAS-28 score in rheumatoid arthritis patients. Significantly higher mean serum 8-OHdG with lower mean serum zinc levels were found in the severe disease activity group (DAS-28 >5.1) when compared to low disease activity (DAS-28 <3.2) (6.72 ± 3.91 ng/mL and 65.4 ± 26.2 µg/dL vs. 5.15 ± 3.45 ng/mL and 76.8 ± 28.8 µg/dL, respectively) (p = 0.02 and p = 0.037, respectively).

**Table 3:** Serum 8-OHdG and zinc level stratified by disease activity. *: Comparing high to low disease activity. 8-OHdG = 8-hydroxy-2-deoxyguanosine; DAS-28 = Disease Activity Score-28

Variable	Disease activity			P-value
	High DAS-28 >5.1 (n = 34)	Moderate DAS-28 3.2–5.1 (n = 91)	Low DAS-28 <3.2 (n = 139)	
8-OHdG (ng/mL) (mean ± SD)	6.72 ± 3.91	5.56 ± 3.81	5.15 ± 3.45	*0.02
Zinc (µg/dL) (mean ± SD)	65.4 ± 26.2	72.5 ± 26.9	76.8 ± 28.8	*0.037

Correlation between 8-OHdG and other parameters in patients and healthy individuals using Pearson’s correlation coefficients (r) are illustrated in Table 4. A negative correlation was found between zinc level and 8-OHdG in the patient group (r = -0.039, p = 0.038) and the correlation was statistically significant but was non-significant in healthy individuals (r = -0.18, p = 0.09). A significant negative correlation was found between DAS-28 and 8-OHdG in rheumatoid arthritis patients (r = -0.26, p = 0.043). A positive significant correlation was found between BMI and 8-OHdG in the healthy individuals group (r = 0.22, p = 0.02).

**Table 4:** Correlation between 8-OHdG and studied parameters in patients and healthy individuals using Pearson’s correlation coefficients (r). 8-OHdG = 8-hydroxy-2-deoxyguanosine; BMI = body mass index; DAS-28 = Disease Activity Score-28

Variable	Rheumatoid arthritis patients		Healthy individuals	
	r	P-value	r	P-value
Age	0.039	0.58	0.09	0.31
BMI	0.029	0.11	0.22	0.02
Zinc	-0.39	0.038	-0.18	0.09
DAS-28	-0.26	0.043	--	--

## Discussion

The most striking finding among patients with rheumatoid arthritis included in this study was high DNA damage. A high DAS-28 score was associated with a low mean serum zinc level and a high degree of DNA damage. When comparing patients with high DAS-28 scores to those with low DAS-28 scores, significantly higher mean serum 8-OHdG levels and significantly lower mean serum zinc levels were found. Moreover, 83.7% of patients with rheumatoid arthritis had a high degree of DNA damage compared to controls, although 28.7% of the controls who were healthy individuals also showed a high degree of DNA damage. Our study results in combination with previous studies from Southeastern Turkey suggest that inflammation is the main process that leads to oxidative stress that ends with oxidative DNA damage [2]. Measuring the serum levels of 8-OHdG is a promising marker that could be used to assess the degree of DNA damage. Our results showed that the degree of oxidative DNA damage was correlated with increasing DAS-28, implying that severe disease leads to more DNA damage and the development of related complications. Our results in combination with another study showed that there is increasing evidence suggesting a close link between inflammatory process, oxidative stress, oxidative DNA damage, and development of complications in rheumatoid arthritis patients as well as the fact that the cells of patients with autoimmune diseases are more sensitive and more prone to genotoxic stress than healthy individuals [14].

This study confirmed that oxidative DNA damage is linked to low serum zinc levels in rheumatoid arthritis patients, which may contribute to the high cellular production of ROS in patients compared to healthy individuals, suggesting that oxidative DNA damage mediated by ROS and inflammation is a pathogenic hallmark [1]. Free radicals can directly cause joint damage by attacking cartilage and its proteoglycan and inhibit its synthesis [15]. In patients with rheumatoid arthritis, oxidative stress through ROS as well as lipid peroxidation end products oxidized low-density lipoprotein-cholesterol, and increasing carbonyl from protein oxidation also causes damage to hyaluronic acid in addition to DNA damage. Furthermore, in patients with rheumatoid arthritis, it has been suggested that there may be an impairment of enzymatic or non-enzymatic antioxidant systems, including low serum zinc levels [1].

Free radicals are produced at a physiological level under normal circumstances by many cellular enzymes [16]. Neutralization of these free radicals is done by the antioxidant activity of many other enzymes [17,18] and some non-enzyme antioxidants including the micronutrient zinc [9].

In this study, serum 8-OHdG exhibited a significant inverse correlation with DAS-28 and serum zinc levels in patients. In healthy individuals, a significant positive correlation was observed between serum 8-OHdG and BMI. These results were similar to those reported by Elham et al. [19].

A beneficial protective effect of zinc supplementation in rheumatoid arthritis patients has been reported by some studies [20]. Others have reported that as age progresses, it causes serum zinc levels to further decrease [21]. In healthy individuals, especially in our region, zinc deficiency is prevalent which may be attributed to many factors such as the consumption of soft drinks and eating rice which contains phytate product that chelates zinc in the gastrointestinal tract and inhibits its absorption. This leads to zinc deficiency in healthy individuals and aggravates this deficiency in patients with chronic diseases such as diabetes mellitus and rheumatoid arthritis [9]. All of the above studies compared serum zinc levels in patients with rheumatoid arthritis and a control group and showed that serum zinc levels decreased in rheumatoid arthritis patients compared to healthy individuals.

## Conclusions

Rheumatoid arthritis patients have more severe oxidative DNA damage than normal individuals. There is a negative significant correlation between the serum 8-OHdG and zinc levels and disease activity based on the DAS-28 score, suggesting that increased oxidative stress may be attributed to low antioxidant status. Antioxidants may be a protective intervention to decrease DNA damage as well as oxidative stress in patients with rheumatoid arthritis.
